# The Influence of Nanobubble Size and Stability on Ultrasound Enhanced Drug Delivery

**DOI:** 10.1021/acs.langmuir.2c02303

**Published:** 2022-11-02

**Authors:** Damien V. B. Batchelor, Fern J. Armistead, Nicola Ingram, Sally A. Peyman, James R. McLaughlan, P. Louise Coletta, Stephen D. Evans

**Affiliations:** † Molecular and Nanoscale Physics Group, School of Physics and Astronomy, University of Leeds, LeedsLS2 9JT, United Kingdom; ‡ Leeds Institute of Medical Research, Wellcome Trust Brenner Building, St James’s University Hospital, LeedsLS9 7TF, United Kingdom; § Faculty of Electronic and Electrical Engineering, University of Leeds, LeedsLS2 9JT, United Kingdom

## Abstract

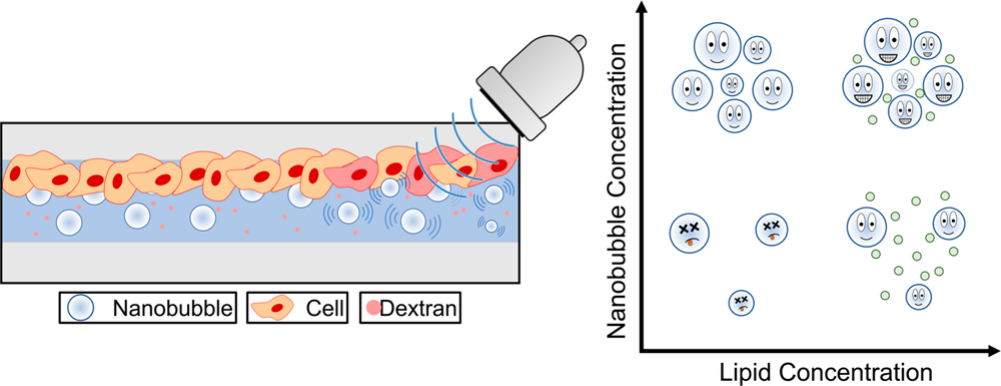

Lipid-shelled nanobubbles (NBs) are emerging as potential dual diagnostic and therapeutic agents. Similar to their micron-scale counterparts, microbubbles (1–10 μm), they can act as ultrasound contrast agents as well as locally enhance therapeutic uptake. Recently, it has been shown that the reduced size of NBs (<1 μm) promotes increased uptake and accumulation in tumor interstitial space, which can enhance their diagnostic and therapeutic performance. However, accurate characterization of NB size and concentration is challenging and may limit their translation into clinical use. Their submicron nature limits accuracy of conventional microscopy techniques, while common light scattering techniques fail to distinguish between subpopulations present in NB samples (i.e., bubbles and liposomes). Due to the difficulty in the characterization of NBs, relatively little is known about the influence of size on their therapeutic performance. In this study, we describe a novel method of using a commercially available nanoparticle tracking analysis system, to distinguish between NBs and liposomes based on their differing optical properties. We used this technique to characterize three NB populations of varying size, isolated via centrifugation, and subsequently used this to assess their potential for enhancing localized delivery. Confocal fluorescence microscopy and image analysis were used to quantify the ultrasound enhanced uptake of fluorescent dextran into live colorectal cancer cells. Our results showed that the amount of localized uptake did not follow the expected trends, in which larger NB populations out-perform smaller NBs, at matched concentration. To understand this observed behavior, the stability of each NB population was assessed. It was found that dilution of the NB samples from their stock concentration influences their stability, and it is hypothesized that both the total free lipid and interbubble distance play a role in NB lifetime, in agreement with previously proposed theories and models.

## Introduction

Nanobubbles (NBs) are rapidly gathering widespread attention in the research community for possible applications in agriculture,^1,2^ water treatment,^3,4^ industrial cleaning,^5^ and biomedicine.^6−8^ Consisting of a low solubility gas core with a stabilizing shell, biomedical NBs, also known as ultrafine bubbles, are <1 μm in diameter, distinguishing them from larger microbubbles (MBs, 1–10 μm), which are currently in clinical use as ultrasound (US) contrast agents.^9^ MB size allows their free flow through the vasculature, while their gas core provides US imaging contrast enhancement owing to the mismatch in acoustic impedance between gas and the surrounding blood and soft tissue. Further, the incident ultrasonic field can induce volumetric oscillations of the MB, leading to increased scattering and improved image contrast.^10,11^ This effect can be amplified by driving MBs at their resonance frequency, which typically occurs within the clinically approved frequency range for diagnostic US (1–20 MHz).^10,12^

MBs have also been widely studied for their potential to enhance drug delivery for treatment of diseases such as cancer.^13−15^ In combination with US, they are capable of locally increasing cell membrane permeability to enhance drug uptake.^14,16−18^ Therapeutic payloads can also be directly incorporated into MBs themselves and their release triggered using high intensity US. The enhanced localized delivery has allowed therapeutic benefit to be observed with lower doses and hence reduced off-site toxicity.^15,19,20^ However, due to their size, MBs are typically restricted to the vasculature, potentially limiting their therapeutic and diagnostic effectiveness.^21,22^

The smaller size of NBs as theranostic agents may be advantageous compared to MBs, as it should allow increased accumulation within the tumor interstitial space and hence potentially reach areas that are inaccessible to MBs.^23,24^ Thus, NBs are thought to have potential for enhanced therapeutic delivery and molecular imaging.^7,25^ Although the validity of the enhanced permeability and retention (EPR) effect has been questioned,^26,27^ several recent studies have highlighted the possibility of NB extravasation, as demonstrated by prolonged wash-out times during imaging or by increased tumor accumulation of fluorescently tagged lipids.^28−31^ Further, Pellow et al.^32,33^ have recently shown the passive extravasation of intact, acoustically active NBs via concurrent acoustic and optical techniques.

The spatial resolution of US imaging is limited by the wavelength of the incident pulse. The use of higher frequency US can improve resolution by shortening length of the pulse train (axial resolution), or by reducing US beam width (longitudinal resolution). As bubble size and resonance frequency follow an inverse relationship, NBs have higher resonance frequencies compared to MBs, and this may allow for increased sensitivity when using high-frequency, high-resolution, contrast enhanced US. However, predicted NB resonance frequencies typically lie well above the frequency range currently used clinically, and the use of higher frequencies will increase acoustic attenuation which may potentially limit their clinical use,^6,34^ although may still be beneficial for preclinical, *in vivo* animal studies. NBs have been utilized for both US imaging and for the delivery of therapeutic agents, using both clinical and preclinical US.^7,35−37^ Recent work by Sojahrood et al.^35,38^ found the resonance frequency to be dependent on driving pressure, attributed to buckling of the lipid shell and a subsequent decrease in surface tension.

NBs are typically produced concurrently with MBs, either by mechanical agitation or microfluidics, and are subsequently separated either by flotation or centrifugation.^39^ This is sometimes followed by a filtration process to remove any remaining larger bubbles; however, this can lead to an adverse effect on NB concentration.^40^ Throughout the literature, the NB isolation techniques, as well as the size of the NBs collected, vary greatly (100–800 nm),^8^ making it difficult to compare their efficacy as vehicles for therapeutic delivery. For example, the shear stress exerted by an oscillating bubble on a cell membrane is nonlinearly proportional to initial diameter. Depending on the model used, an 800 nm diameter NB will produce shear stresses between 10^2^ and 10^4^ times greater than that of a 100 nm NB, assuming both are driven on resonance.^41^ Additionally, for bubbles undergoing inertial cavitation, the velocity of the produced microjet directed toward a nearby cell membrane would be expected to increase by a factor of ∼10^2^.^42,43^

One challenge associated with NBs is accurate determination of their size and concentration. Due to their submicron nature, typical sizing techniques used to characterize MBs (brightfield microscopy, Coulter counters) are unsuitable. Light scattering techniques such as dynamic light scattering (DLS) and nanoparticle tracking analysis (NTA) are routinely used to determine nanoparticle size by observing the Brownian motion of particles as a function of time. However, a typical lipid-coated NB sample will contain a mixed population of aqueous filled liposomes and gas-filled NBs, which these techniques do not overtly distinguish. This, coupled with classical bubble theories that predict NB lifetimes should be on the order of milliseconds,^44−46^ has led to some skepticism in the research community over the existence of stable NBs. However, the recent advent of resonant mass measurement (RMM) has been successful in characterizing stable NBs, in which positively and negatively buoyant populations can be measured separately (i.e., NBs vs liposomes). RMM is suitable for bubbles with sizes >200–300 nm; however, the use of pressure pumps (∼30–70 kPa) to induce fluid flow through the cantilever mechanism may lead to either dissolution of bubbles into the ambient phase or to a phase change of gas to liquid and, hence, potentially underestimate of the number of buoyant particles. Recently Midvedt et al.^47^ developed a holographic NTA system, in which the an interference pattern is created by combining the scattered light and that of an unobstructed reference beam. From this, the phase contrast of each particle is used in addition to their Brownian motion, enabling determination of particle size and refractive index, and as such was able to distinguish NBs from a bulk population. Although still in a preliminary phase, another method that holds promise for NB size characterization was described by Moore et al.^48^ utilizing high-frequency (200 MHz) M-mode imaging to observe stochastic motion of NBs within an agarose gel with known pore sizes. Similar to DLS, autocorrelation of the intensity signal can be used then to estimate NB size.

In this paper, we used an NTA system (NS300, Malvern Panalytical, UK) to distinguish between liposomal and NB subpopulations in a NB sample. We then used this method, in combination with DLS and optical microscopy, to characterize three NB populations of varying mean size, isolated via centrifugation. These NBs were used to investigate the dependence of NB size on their ability to enhance intracellular uptake using clinically relevant US frequencies, by observation of the uptake of fluorescent dextran into SW480 cell monolayers cultured in a microfluidic device. These results did not follow the expected trend, i.e., larger NBs inducing increased uptake. It was postulated that the reason for the unexpected behavior might lie in the NB stability, and thus, the mechanisms behind this, specifically the influence of free lipid concentration and interbubble spacing on NB lifetime, were investigated.

## Materials and Methods

### NB Preparation, Production, and Isolation

An initial MB/NB suspension was prepared using 95:5 molar ratio of the lipids 1,2-dipalmitoyl-*sn*-glycero-3-phosphocholine (DPPC) and 1,2-distearoyl-*sn*-glycero-3-phosphoethanolamine-*N*-[methoxy(polyethylene glycol)-2000] (DSPE-PEG2000) to form the stabilizing shell. Lipids were initially dissolved in 50:50 chloroform:methanol solution, and the solvent removed under nitrogen for ∼60 min, followed by vacuum desiccation overnight. The resultant lipid film was then rehydrated with PBS containing 1% (v/v) glycerol, by stirring and heating at 55 °C for 20 min, to a final lipid concentration of 2 mg/mL. The lipid solution was then tip sonicated (20 kHz, 150 W, Sonifier 250, Branson, USA) for 40 min at 4 °C to produce small lipid vesicles (∼100 nm). This solution was then centrifuged at 17,000*g* for 30 min and aspirated, first to remove any titanium deposited during the tip sonication process and second to ensure the absence of any large lipid aggregates.

To produce the initial bubble solution, 1 mL of vesicle solution was added to a 1.5 mL glass vial, and the solution and vial headspace was saturated with perfluoropropane (C_3_F_8_) gas, maintaining a gas pressure of 300 mbar for 2 min. Gas flow was controlled using a p-pump (Mitos P-pumps, Dolomite, UK) and a PC using the Dolomite Flow Control Centre.^49^ The vial lid was then replaced and sealed with parafilm, prior to mechanical agitation for 45 s (VialMix, Bristol Myers Squibb, USA).

To isolate NBs, this solution was then added to 9 mL of PBS in a 15 mL centrifuge tube and centrifuged RCFs of 100, 500, or 1000*g*, due to the relationship between NB size and terminal flotation velocity (eq 1):
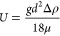
1where *U* is the terminal flotation velocity (m/s), *g* is the gravitational acceleration (m/s^2^), *d* is the diameter (m), Δρ is the difference in density between the medium and the core (kg/m^3^), and μ is the dynamic viscosity of the fluid, which here is water (8.9 × 10^–4^ Pa s).

Post-centrifugation, the NBs were isolated by removal of the lower 6 mL of solution using a long, fine needle (19 g × 2.0″, Terumo) and 5 mL syringe (total volume >6 mL), taking care to avoid cross-contamination of the NB sample and MB foam layer.

### NB Population Characterization

To determine the size and concentration of submicron bubble populations, the light scattering techniques of NTA (size and concentration) and DLS (size only) were used.

#### Nanoparticle Tracking Analysis

For NTA measurements (NanoSight NS300, Malvern Panalytical, UK), NB samples were measured at between 1 and 50× dilution in PBS, depending on the initial sample concentration, such that recorded videos were at optimal particle concentration for particle tracking (i.e., ∼10^8^–10^9^ particles/mL). Samples were illuminated with a 488 nm laser, and individual particles were tracked and analyzed using NTA 3.3 software. During data acquisition, the camera level was set to between values of 3 and 4, such that highly scattering particles (i.e., NBs) were detected, but particles with a lower scattering intensity (i.e., lipid vesicles) were not.

Measurements consisted of 5 × 60 s videos, between which the sample was advanced to observe and track a unique set of particles. Each video was postprocessed using a software detection threshold of 20, and the mean and standard error for each sample were calculated.

Validation of the NTA system was performed using monodisperse NIST standard polystyrene beads with calibrated diameters of 620 ± 24 nm and 788 ± 26 nm (Figure S1), in which NTA measured modal sizes of 526 and 711 nm, respectively. The two samples had stated concentrations of 3.7 × 10^11^ /mL and 7.7 × 10^11^ /mL. Values acquired by the NTA system were (3.5 ± 0.1) × 10^11^ /mL and (6.0 ± 0.4) × 10^11^ /mL, respectively, close to the nominal values given by the supplier.

#### Dynamic Light Scattering

DLS (Zetasizer NanoZS, Malvern Panalytical, UK) measurements were conducted using NBs at their initial concentration post isolation. Samples were illuminated with a 633 nm laser and backscattered light detected at an angle of 173°. Distributions shown are based on an intensity distribution due to NB samples containing a mixed population with different optical properties, and meaning the number and volume weighted distributions cannot be accurately calculated. As such, the intensity-weighted sizing data will be biased toward larger particle sizes and those with a larger change in refractive index from the medium (i.e., bubbles).

#### Brightfield Microscopy

Brightfield microscopy was used to determine the concentration of optically visible bubbles (OVBs) in NB samples. 30 μL of sample was introduced into a 50 μm depth chamber on a glass slide, and OVBs allowed to rise for 5 min to ensure they were all in the same focal plane. An inverted microscope (Nikon 90i, Japan) was used to image the bubbles with a 40× objective (NA = 0.6) and a CCD camera (DS-Fil 5Mega pixel, Nikon, Japan) was used to take 10 images for each sample. Due to the resolution limit of the microscope (∼600 nm, 0.16 μm/pixel), determination of NB size from optical imaging is not possible. However, due to their strong light scattering, it is still possible to detect and count these bubbles. Image analysis was performed using a custom ImageJ script, to determine the total number of particles in each image, and then converted to a concentration value. Using this system, the lower limit of detectible bubble concentration is ∼10^6^ /mL, assuming one bubble per image.

### Cell Culture and On-Chip Culture

The SW480 colorectal cancer cell line was provided by European Collection of Authenticated Cell Cultures. Cells were cultured in Dulbecco’s modified Eagle medium (DMEM/F-12; Gibco, USA) supplemented with 10% fetal bovine serum and 2 mM GlutaMax. Passage numbers were kept below 50 for all experiments. Cells were detached by incubation with TrypLE (Thermo Fisher Scientific) for 5 min and counted using a hemocytometer. The cell suspension was adjusted to a concentration of 7 × 10^5^ cells/mL, and 30 μL of this suspension pipetted directly into the microfluidic channels. For uptake studies, cells were seeded onto a microfluidic device (μ-Slide VI^0.4^, iBidi, Germany). Each microfluidic device consisted of 6 individual channels with a channel height of 0.4 mm, length of 17 mm, and width of 3.8 mm. Channels were pretreated with iBiTreat for culture of adherent cell lines. Devices were then inverted such that cells adhered to the top of the microfluidic channel. After 2 h, the devices were righted, and 60 μL of DMEM was added to each reservoir simultaneously. Cells were cultured on-chip for 48 h prior uptake studies. During on-chip culture, the bottom surface of the device was raised above the incubator surface to allow air flow and promote gas exchange.

### Acoustic Set Up and Ultrasound Exposure

**Figure 1:**
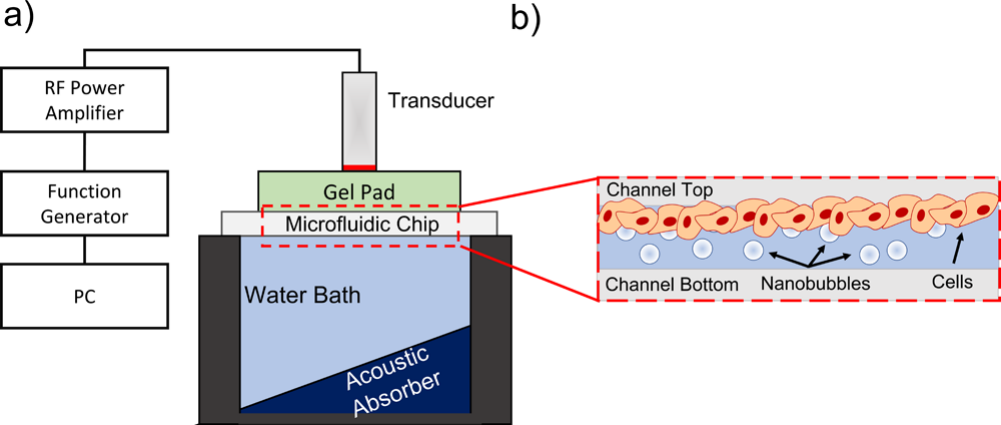
(a) Schematic showing the acoustic and microfluidic setup used for sonoporation experiments. The transducer was driven by a +53 dB power amplifier, and a computer-controlled function generator was used to provide sinusoidal burst cycles. The transducer was coupled to the top of the microfluidic chip using a gel stand-off pad, positioned above a water bath with an acoustic absorber positioned at a 45° angle. (b) Schematic showing cells cultured on the top surface of the microfluidic channel to allow the intrinsic buoyancy of the bubbles to bring them in direct contact with cells (not to scale).

An unfocused, 2.25 MHz central frequency transducer (V323-SM, Olympus, USA) with an element diameter of 6.35 mm was used for sonoporation studies. The transducer was driven by a +53 dB power amplifier (A150, E&I Ltd., USA), and a computer-controlled function generator (TG5011A, Agilent, USA) was used to provide sinusoidal burst cycles to the amplifier. Free-field pressure of the transducer was determined using a needle hydrophone (0.2 mm, Precision Acoustics Ltd., UK), calibrated by the National Physics Laboratory (Middlesex, UK). Each US exposure consisted of the following parameters: Driving frequency = 2.25 MHz, peak negative pressure = 900 kPa, mechanical index = 0.6, pulse repetition frequency = 1 kHz, duty cycle = 1%, and total duration = 5 s. The US transducer was coupled to the top of the microfluidic chip using a 20 mm thick gel stand-off pad (AquaFlex, Parker, USA) ensuring the channel was situated in the far-field of the US beam.^14^ At this distance, the predicted beam width can be estimated from eq 2, where α is the half-angle beam spread, *k* is a constant, *c* is the speed of sound (m/s), *f* is the frequency (MHz), and *D* is the element diameter; *k* is a constant depending on the point that the beam spread is calculated. For −6 dB (i.e., 50% reduction) and for −20 dB (90% reduction), *k* = 0.56 and 1.08, respectively.^50−52^
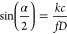
2Assuming the speed of sound to be 1480 m/s (i.e., in water), element diameter of 6.35 mm, the predicted beam width is predicted to be 2.3 mm and 4.5 mm for −6 dB and −20 dB, respectively. The microfluidic chip was positioned above a water bath with an acoustic absorber positioned at a 45° angle to reduce acoustic reflections and the formation of standing waves (Figure 1a). However, based on 2D *k*-wave simulations, the maximum pressure may be expected to reach up to 2000 kPa at the cell monolayer (Figure S2).

### Ultrasound Enhanced Drug Delivery

For the uptake studies, a red fluorescence probe (70 kDa Texas-Red dextran, ThermoFisher) was used to quantify uptake, while a green fluorescence live stain (CellTracker Green CMFDA) was used to determine cell viability post treatment.^18^ NB samples were prepared as described above and mixed with TexasRed-dextran to reach the desired NB concentration and a final dextran concentration of 14 μM. For control samples (no treatment and US only), PBS containing 14 μM dextran was used. NBs were then added to the microfluidic channels by pipetting 60 μL of sample directly into a reservoir, and then withdrawal of 60 μL from the opposing reservoir. This was repeated in triplicate to ensure the channel contained only NB solution. Chips were then covered with foil and left for 60 min at 21 °C to allow NBs to rise to the top of the channel and hence be in proximity with the cell monolayer (Figure 1b).

The effect of changing the length of this incubation period is shown in Figure S3. For all treatment conditions, the adjacent channel was left blank to avoid cross over between the US beam. Channels were then exposed to US (where appropriate) and left for a further 10 min to promote uptake. Channels were then washed with DMEM (5 × 100 μL), followed by 2 μM CellTracker Green (5 × 100 μL), and incubated at 37 °C for 30 min. Channels were then washed with DMEM (5 × 100 μL) prior to confocal fluorescence imaging.

### Confocal Fluorescence Imaging

Microfluidic chips were imaged using a laser scanning confocal microscope (Leica DMi8/SP8) to determine the location of live cells and dextran fluorescence. During the cell culture process, a small proportion of cells did not successfully adhere to the top of the channel and continued adhered culture on the bottom of the channel. Due to the confocality of the microscope (1 airy unit), it was possible to image exclusively cells adhered to the top of the microfluidic device, and hence exposed to NBs. Images were taken in sequential mode using and 488 and 532 nm laser with emission windows of 493–749 nm and 557–781 nm, corresponding to the CellTracker Green and TexasRed-dextran, respectively. These values were determined by the in-built DyeAssistant software to maximize fluorescence intensity and minimize cross talk.

Fluorescence and brightfield maps of each microfluidic channel were taken using the TileScan feature, consisting of multiple images (512 × 512 px) which were then combined to create the final image. The autofocus setting was used in between each image location, determining the focal plane with the maximum intensity in the green fluorescence channel across 5 steps within a user-centered 60 μm window. During imaging, devices were maintained as 37 °C (iBidi heating system, iBidi, Germany).

### Fluorescence Image Analysis and Quantification of Uptake

Confocal fluorescence maps were analyzed using image processing tools in MATLAB (2019b, Mathworks Inc., USA) to determine the dextran fluorescence intensity inside live cells. An image mask was generated from the CellTracker Green image and then applied to a background subtracted dextran image. The image mask was generated by binarization of the original CellTracker Green image and by morphologically opening and dilating the binary image, to remove noise and ensure the entirety of each cell was included. As the US exposure is constrained to a discrete region in the center of the microfluidic channel, an average red fluorescence profile in the *x*-direction along the chip was calculated to identify this region. The fluorescence profile was smoothed using a Savitzy–Golay filter (2 mm window) followed by a baseline subtraction.^53^ From these profiles, the total fluorescence intensity (TFI) was quantified. Initially, a Gaussian distribution was fit to the smoothed data to determine the peak center, *x*_c_, and standard deviation, σ. If successful, the background subtracted, nonsmoothed profile was integrated between (*x*_c_ - 2σ) and (*x*_c_ + 2σ) to determine the total intensity. If the fit was unsuccessful (i.e., no clear uptake detected), then the data were integrated across a 7 mm window situated in the center of the profile. A detailed description and example of the analysis are shown in Figure S4.

## Results and Discussion

### Nanobubble Isolation and Characterization

MBs and NBs were initially produced via mechanical agitation, and NBs of different sizes were then isolated via centrifugation at three different relative centrifugal forces (RCFs). NB populations were then characterized to determine size (DLS, NTA) and concentration (NTA), while the presence of potentially larger optically visible bubbles (OVBs) was subsequently determined using brightfield microscopy.

**Figure 2:**
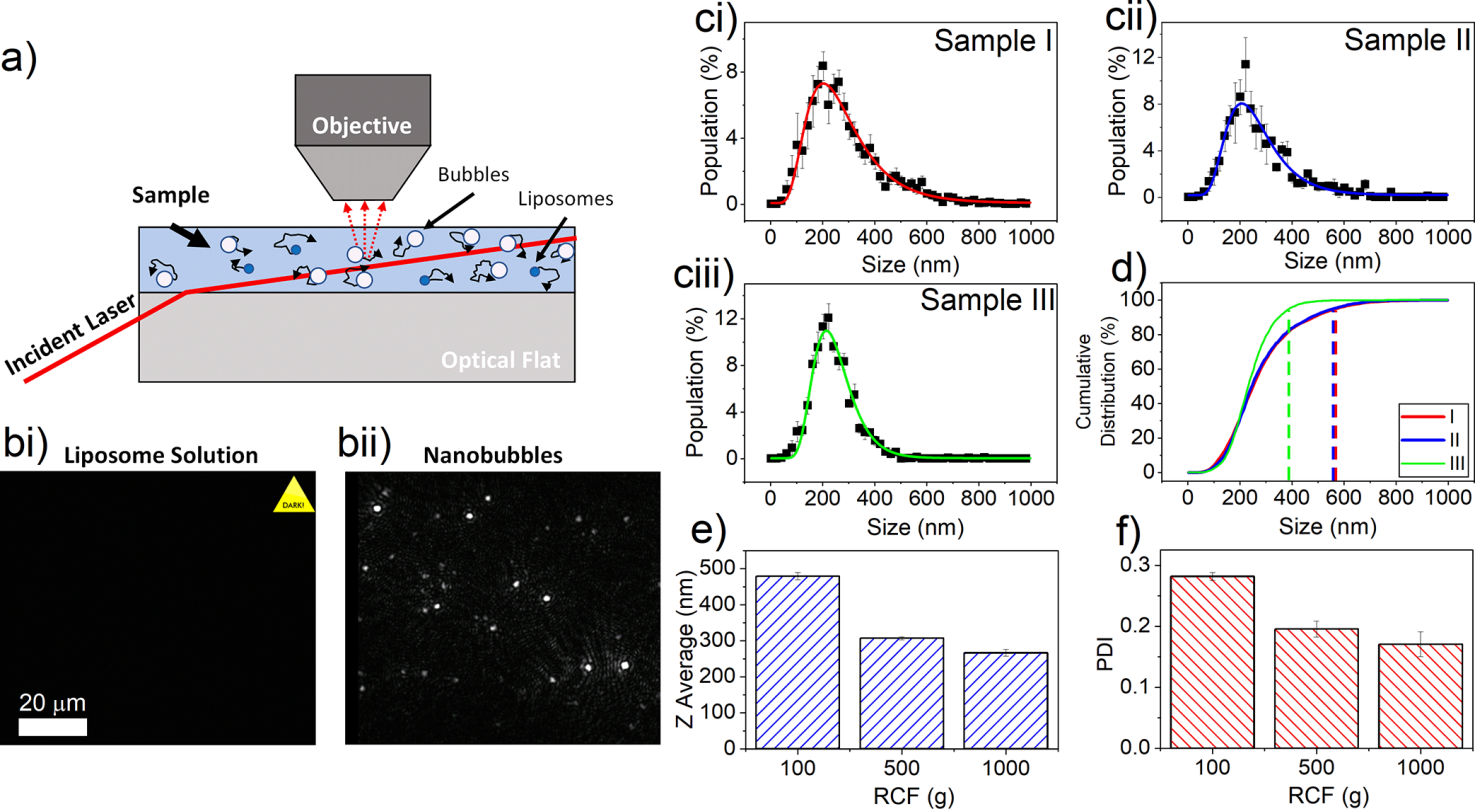
Size and concentration characterization of NBs using NTA and DLS. (a) Schematic showing the working principle of NTA, in which the random walk of nanoparticles is tracked by observing light scattered by individual particles. (b) Images collected during NTA data acquisition, demonstrating that at the same total lipid concentration and camera acquisition settings the liposome precursor solution (bi) is not visible, whereas after NB production the NB are clearly visible and detected by the NTA software (bii). (c) NTA measured populations for samples separated via centrifugation at 100*g* (ci), 500*g* (cii), and 1000*g* (ciii). For clarity, only every 4th data point is shown. (d) Cumulative distribution for each NB sample, displayed as percentage of the population. Dotted lines represent that 95th percentile for each population. (e) DLS *Z*-average size and (f) DLS PDI of NB populations.

Figure 2a shows a schematic of NTA, in which a sample is illuminated, and the scattered light used to track the random walk of individual particles in the plane of illumination and hence determine their size. During NTA measurement, a parameter known as “camera level” is adjusted, influencing image brightness by varying the camera gain, shutter time, and upper limit of the intensity histogram. At lower camera levels (<10), the image brightness is varied by increasing the camera shutter time. At higher camera levels (≥10), a combination of shutter time, camera gain, and upper limit of the intensity histogram are adjusted (maximum value of 16380). Full details of these parameters for each camera level are given in Table S1. A typical NB sample is expected to contain a mixture of both gas-filled bubbles and aqueous-filled liposomes of similar size, and as such, it can prove difficult to distinguish between these populations. For NB and liposome samples at the same dilution and acquisition settings (camera level 12, camera gain = 146 au, shutter time = 30 ms, histogram upper limit = 11529, lipid concentration = 2 μg/mL), samples are nearly indistinguishable using NTA (Figure S5). For both samples, modal size was 90 nm, and concentrations (2.3 ± 0.1) × 10^12^ /mL and (3.2 ± 0.1) × 10^12^ /mL for liposomes and NBs, respectively. However, by utilizing a low camera level setting, compared to typical NTA measurement settings, we can determine the NB subpopulation, such that the liposome population is not visible. At camera levels used to identify NBs (3–5), the image brightness is increased by an increasing in the shutter time (0.33, 0.58, and 1.13 ms for camera levels 3, 4, and 5, respectively), while the gain remains constant (15 au). The intensity of scattered light from small particles can be described nominally by Rayleigh scattering for small particles, when *x* ≪ 1, where *x* = 2π*r*/λ, where *r* is particle radius and λ is wavelength of light. The relationship between scattering cross section, σ_s_, and the relative refractive index between the medium (*n*_water_ = 1.33) and the particle, *m*, is described by eq 3.^54^ Hence, a 100 nm NB (*n*_air_ = 1) would be expected to scatter approximately 40× more light than an equivalent liposome (*n*_liposomes_ = 1.38).^55^
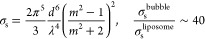
3where σ_s_ is the scattering cross section, *d* is particle diameter (m), *m* is the relative refractive index (*n*_particle_/*n*_medium_), and λ is the incident light wavelength (m).

Due to their higher relative scattering, NBs can be detected at a lower camera level compared to liposomes of similar size, allowing for measurement of NBs exclusively. Figure 2b shows images of liposomes and NBs acquired with the same settings (camera level 3–5). Liposomes present in the NB precursor solution are not visible in Figure 2bi, and NBs are clearly visible in Figure 2bii. NTA images of a liposome only sampled over a range of camera levels are presented in Figure S6 and show that liposomes are not detectable until camera level >8 (camera gain = 15 au, shutter time = 7.93 ms, histogram upper limit = 16380). For NBs, it is also observed that following exposure to high intensity focused ultrasound (HIFU), the NB concentration decreased 10-fold from (3.0 ± 0.3) × 10^9^ /mL to (2.9 ± 0.5) × 10^8^ /mL (Methods S1). The latter results supports the suggestion that the increased scattering intensity observed in the NTA measurements can be attributed to acoustically active, gas-filled particles. Further, recent publications utilizing a similar NB production method and resonant mass measurement demonstrate the presence of submicron buoyant particles.^56,57^

Figure 2c shows the NTA data measured from populations of NBs isolated via centrifugation at (ci) 100*g*, (cii) 500*g*, and (ciii) 1000*g* as well as their cumulative distributions shown in (d). For convenience, we refer to these samples as I, II, and III, respectively. As the RCF is increased, the modal size of the population remains constant (Table 1). However, the proportion of larger bubbles decreases, signified by a decrease in the observed mean size and as shown in the cumulative distribution plot (Figure 2d), as progressively smaller bubbles are removed during the separation process. For each sample (I, II, and III), the 95th percentile for size was 568 nm, 558 nm, and 388 nm, respectively. This is also highlighted by volume-weighted NTA distribution plots (Figure S7). The NB concentration was found to decrease with increasing RCF, from 4.9 ± 0.5 × 10^10^ /mL for sample I to 0.9 ± 0.1 × 10^10^ /mL for sample III. The NTA data show that the gas volume fraction decreased with increasing RCF and was 0.11, 0.07, and 0.01%, for samples I, II, and III, respectively. The results in Figure 2c,d shows that few NBs > 600 nm were detected using NTA, and hence, it may be assumed that all bubbles in the sample are NBs (<1 μm). The limit of detection of the NTA system (i.e., lowest measurable NB size) appears to be ∼100 nm, and it is possible that additional undetected, smaller NBs exist below this limit. This limit of detection is similar to that found in other techniques (such as RMM) that can distinguish between bubbles and nonbubbles.^56^

**Table 1:**
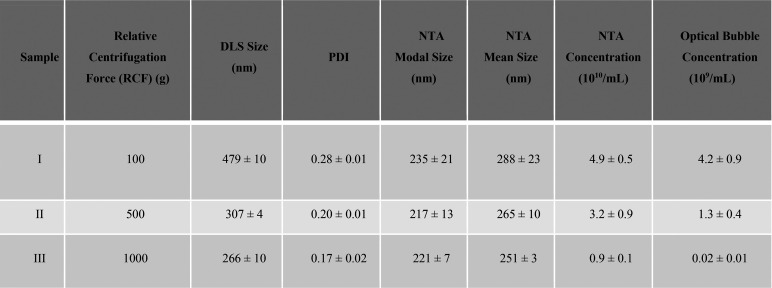
Summary of the NB Populations Isolated via Centrifugation at 100*g*, 500*g*, and 1000*g*^a^

a NBs were characterized via DLS (size), NTA (size and concentration), and optical microscopy (concentration of OVBs).

As NTA is limited to the analysis of submicron particles, populations were also measured using DLS in which the bulk sample is analyzed, as opposed to single particle tracking (Figure 2e,f), and also has a larger measurable size range (10–10,000 nm) and can be performed at relatively high bubble concentrations (i.e., undiluted NB samples). While DLS cannot distinguish between NBs and liposomes, it is expected that the NBs will dominate the scattering intensity compared to liposomes due to their increased size and scattering cross section (eq 3). DLS displayed a concurrent decrease in both *Z*-average size and polydispersity index (PDI), as more of the larger bubbles are removed with increasing RCF.

Brightfield microscopy was used to determine the concentration of any optically visible bubbles (OVBs). Since the resolution limit of the microscopy system used was ∼600 nm (NA = 0.60), accurate sizing of bubbles ≤1 μm was not possible, and hence, this method was used purely to determine concentration of OVBs. The term OVB here refers to any bubbles which were visible on our microscopy system (40× objective), either by the resolution of individual bubbles or by visualizing scattered light from bubbles below the resolution limit. However, it is not clear what size the smallest optically observable bubbles are, due to the relationship between particle size and scattering cross section (eq 3). Prior to centrifugation, OVB concentration was ∼5 × 10^10^ /mL, and OVB concentrations postcentrifugation are shown in Table 1. An example image of OVBs present in each sample (I, II, and III) is shown in Figure S8. OVB concentration decreases with increasing RCF, and for sample III, OVBs are nearly completely removed from the sample (2 ± 1 × 10^7^ /mL), decreasing by a factor of ∼200 compared to sample I. Comparatively the concentration of NBs determined by NTA decreased only by a factor of 5, suggesting the preferential removal of larger bubbles with increasing RCF. Further, the concentration of bubbles measured by NTA is an order of magnitude greater than those detected optically, highlighting that a combination of light scattering techniques and optical microscopy is currently required to confirm both OVB and NB populations within a sample.

### Effect of NB Size on Drug Uptake

**Figure 3:**
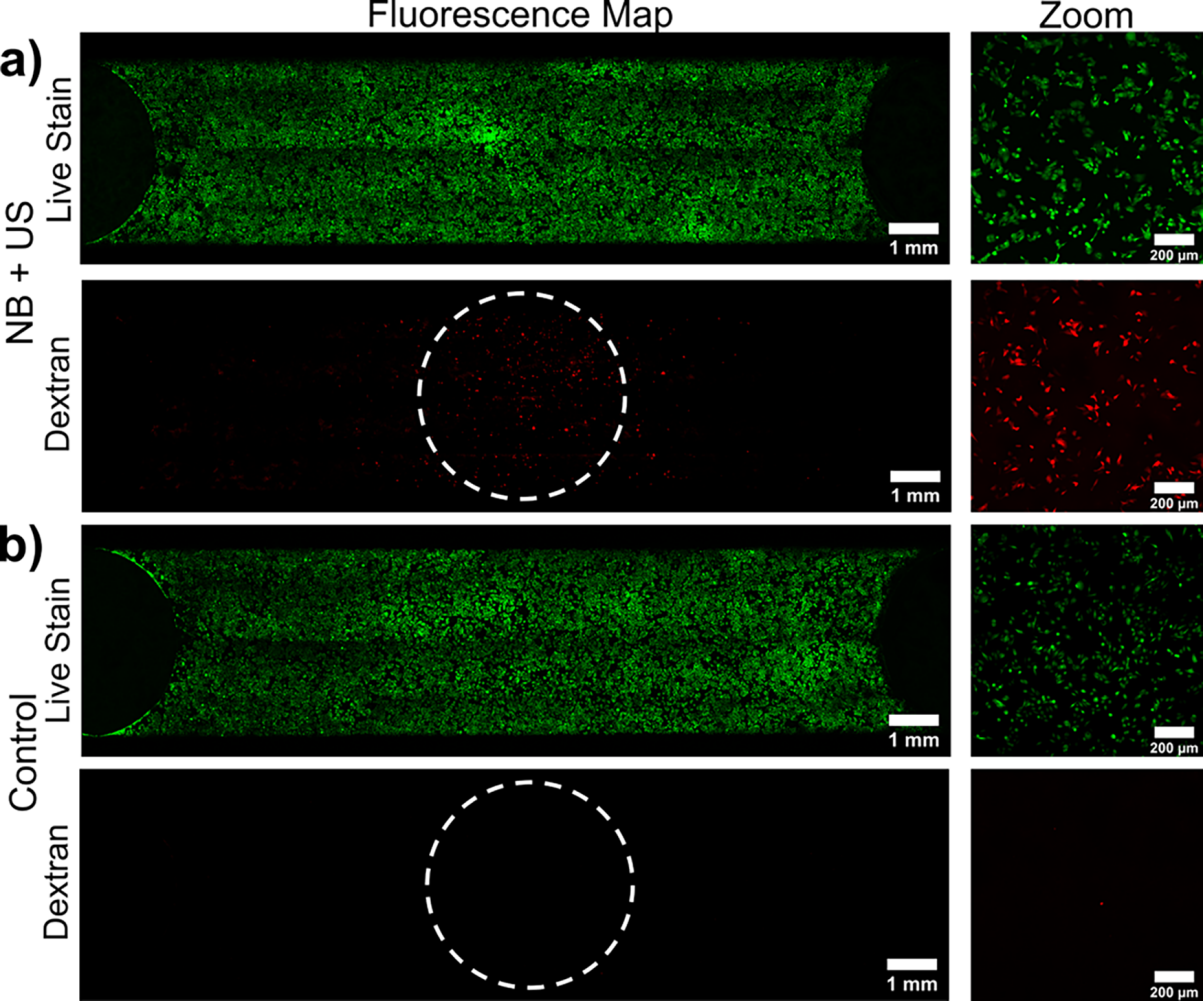
(a) Confocal fluorescence images showing live stain (green) and 70 kDa TexasRed-dextran fluorescence (red) emissions from monolayers of SW480 cells. Images show cells after treatment with NBs and US (MI 0.6, *f*_0_ = 2.25 MHz, PRF = 1 kHz, duty cycle = 1%, total duration 5 s). (b) A control sample of US only. Full fluorescence maps of the microfluidic chip were used for quantitative analysis of uptake, and the increased “zoom” images show clear colocalization of fluorescence between dextran and the live stain indicative of dextran uptake in live cells. The white dashed circle represents the −20 dB point of the pressure field.

NBs in combination with US have been shown to locally increase cellular uptake of small molecules, such as chemotherapeutics and model drugs.^25,58^ Here, we investigate the effect of NB size and concentration on the enhancement of drug uptake using clinically relevant US. A monolayer of SW480 cancer cells was cultured within a microfluidic device and exposed to a combination of US (MI 0.6, 2.25 MHz, PRF = 1 kHz, duty cycle = 1%, 5 s total duration) and NBs. Fluorescently tagged dextran (TexasRed-dextran, 70 kDa) was codelivered at a concentration of 14 μM as a model drug to identify US and NB mediated uptake via confocal fluorescence imaging. These US parameters were based on similar treatments developed by our group recently, in which the efficacy of therapeutic MBs were assessed in both *in vitro* 3D cell culture models^14^ and *in vivo* murine models.^15,59^ The choice of fundamental frequency and MI also provides clinical relevance, especially through the use of NBs which typically have a resonance frequency greater than the clinically approved range (>20 MHz).^60^ For each treatment condition, a fluorescence map of the cell monolayer across the microfluidic chip was used to quantify the dextran uptake, by measuring the fluorescence intensity of dextran inside live cells within the insonated region. Figure 3 shows images taken after treatment with NBs + US (sample I, 4 × 10^10^ NBs/mL), and a control sample treated with US only. After treatment with NB + US, fluorescence images show an increase in dextran fluorescence localized within the central, insonated region of the microfluidic chip (Figure 3a). “Zoom” images taken at increased magnification show a clear colocalization of both dextran and the live stain, and hence uptake in live cells. This is compared to a control sample (US only, Figure 3b), where no significant fluorescence was measured across the chip, in addition there is no fluorescence colocalization.

**Figure 4:**
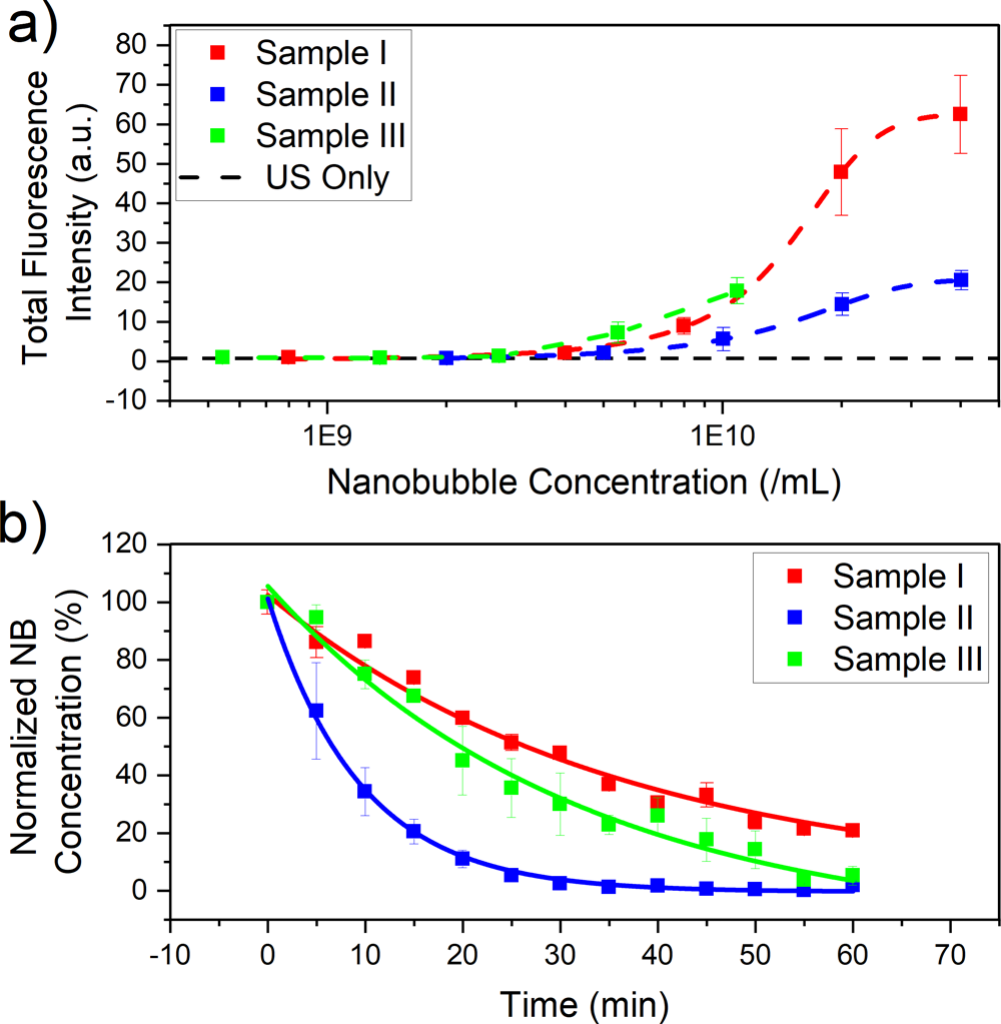
(a) TFI inside live cells due to US and NB mediated uptake of 70 kDa TexasRed-dextran (14 μM), determined via confocal fluorescence microscopy for three NB samples isolated via centrifugation at RCF = 100*g*, 500*g*, and 1000*g* (samples I, II, and III, respectively). (b) Normalized NB concentration measured over a 60 min period at a matched initial NB concentration of 1 × 10^9^ bubbles/mL. The data are fitted to an exponential decay function. Error bars represent the standard error across a minimum of 3 experiments.

US/NB mediated uptake was quantified for each NB sample (I, II, and III) across a range of NB concentrations, by determining the TFI of dextran inside live cells. Figure 4a shows the TFI plot against the concentration NBs were initially delivered on chip. Control chips of either no treatment or US only had TFIs of 0.92 ± 0.62 au and 0.66 ± 0.64 au, respectively. Samples I, II, and III each demonstrated enhanced dextran uptake at their highest NB concentration, which for samples I and II was ∼4 × 10^10^ NBs/mL and for sample III ∼1 × 10^10^ NBs/mL. For all samples, decreasing NB concentration led to a corresponding decrease in TFI (dextran uptake), indicating NB concentrations of >5 × 10^9^ NB/mL are necessary to see any detectable increase in dextran uptake. At matched NB concentration, insonation of sample I leads to increase uptake compared to sample II. However, at matched NB concentration of 1 × 10^10^ NBs/mL, sample III outperforms sample II (TFI = 17.8 ± 3.3 compared to 5.6 ± 2.9). In work by Pellow et al., it was found that for NBs of similar size (200–400 nm) at the pressures used in our study (900 kPa, MI = 0.6), NBs would be expected to produce fundamental, subharmonic, ultraharmonic, and broadband emissions. Hence, in our on-chip study, we would expect NBs to exhibit a combination of both stable oscillation (microstreaming) and inertial cavitation (microjetting), which may induce intracellular uptake. It should be also noted that the concentration of NBs required to induce observable dextran uptake are several orders of magnitude higher than that of MBs utilized *in vitro*,^14,18^*in vivo*,^15,59^ or in clinical trials.^61^ However, the yield concentration of NBs is intrinsically higher than that of MBs.

During these experiments, NBs are left on-chip for 60 min prior to insonation to come into the vicinity of the cell monolayer via flotation. As such, the actual NB size distribution and concentration at the top of the channel at the time of insonation will be different to that when NBs are initially homogeneously distributed throughout the channel. Predictions of this may provide an explanation to why sample III outperformed sample II at matched initial NB concentrations. As NB terminal rise velocity is proportional to *d*^2^ (eq 1), the population of bubbles that are in contact with the cell monolayer, and hence primarily contribute to uptake, is biased toward larger NBs in the population. In fact, all bubbles >458 nm in diameter would be expected to have risen a total distance of at least 400 μm and hence contribute to enhanced uptake. In the three NB samples (I, II, III), the majority of NBs are smaller in diameter than this threshold (88.2, 88.9%, and 98.6% respectively). Hence, the influence of the flotation time on NB population is associated only with small increases to the modal size of each population (302 nm, 299 nm, and 260 nm) and cannot explain the observed behavior of samples II and III (Figure S9). Furthermore, the absolute concentration (or density) of NBs present at the top of the microfluidic channel (i.e., in close vicinity to the cell monolayer) should be considered for proper comparison between samples. Predicted NB surface densities, assuming perfect stability, ranged between 0.1 and 10 NBs/μm^2^ (Figure S10) and showed that sample III outperforms samples I and II at matched surface density and coverage. Based off of these calculations, it is evident that the ability of each sample to enhance dextran uptake is not solely linked to their size, or density, at the cell monolayer. However, the previous analysis assumed the ideal case of perfect NB stability.

NB stability was measured to determine whether this influencing the observed NB performance. As NB stability is not easily measurable on-chip, NB concentrations were measured *in situ* in the NTA system to mimic the on-chip conditions, over a 60 min period at an initial concentration of ∼10^9^ NBs/mL, corresponding to an optimal concentration for the NTA analysis.

Figure 4b shows the normalized NB concentration over time. The populations decay exponentially with half-life’s, τ_1/2_, of 31.8 ± 6.2, 8.6 ± 0.4, and 19.6 ± 1.9 min for samples I, II, and III, respectively. This trend in stability (i.e., sample III having enhanced stability compared to II) may explain why sample III had increased uptake enhancement compared to sample II.

### Investigation into NB Stability

While there is no universally agreed theory for NB stability, the Laplace pressure, Δ*P*, predicts that bubble stability decreases with bubble radius, *r*, (, where σ is surface tension). However, results in Figure 4b show that sample III exhibit increased stability compared to II, with sample I being the most stable. It was also found that the average (mean and mode) size of all samples remained constant over the 60 min period (Figure S11). This may suggest that in bubbles there is an occurrence of rapid bubble dissolution or that NBs coalescing and no longer within the measurable size range of NTA (i.e., >1000 nm). However, the latter is unlikely, as this would require the coalescence of ∼10^2^ NBs.

**Figure 5:**
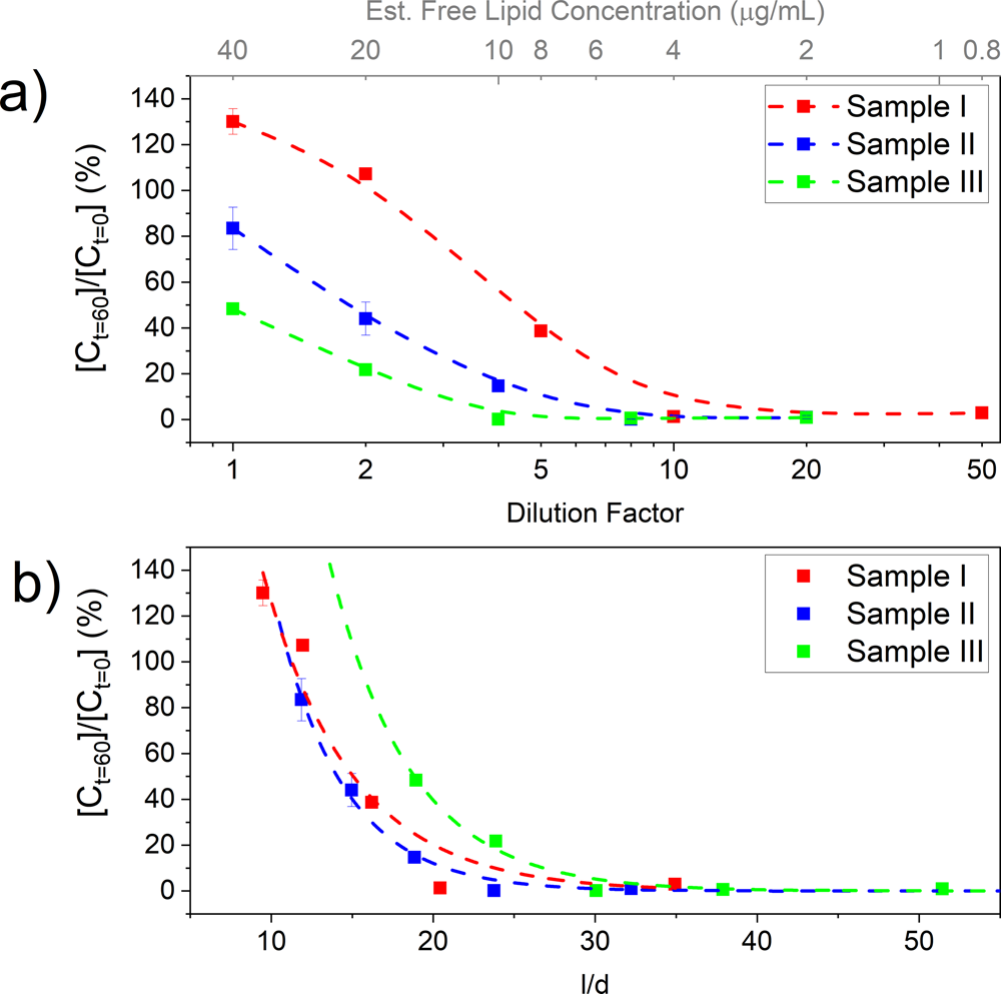
Stability of NBs samples I, II, and III as a function of dilution from their initial, as-prepared concentration. NB concentration was measured initially at *t* = 0 min and at *t* = 60 min after storage at varying dilutions. The ratio of this final concentration [*C*_*t*=60_] to the initial concentration [*C*_*t*=0_] was then used to quantify stability when stored at different dilutions. (a) [*C*_*t*=60_]/[*C*_*t*=0_] for dilution factors ranging from 1 (stock concentration) to 50 (i.e., 50× diluted). The top *x*-axis also shows the estimated free lipid concentration in the NB sample, assuming an initial lipid concentration of 40 μg/mL in an undiluted sample. Data are fit with a spline curve to guide the eye. (b) [*C*_*t*=60_]/[*C*_*t*=0_] as a function of the ratio of the interbubble distance, *l*, and mean NB diameter, *d*, achieved by dilutions.

Although the measurements in Figure 4b were performed at matched NB concentration, the differing initial NB concentrations for each sample (Table 1) meant that the sample dilution required to reach a set NB concentration varies between samples. For example, a dilution of ∼10× was required for sample III compared to ∼30× for sample II and ∼50× for sample I, to reach the matched NB concentration of 10^9^ NBs/mL used in the *in situ* stability measurements. As sample III demonstrated increased stability compared to II, it may suggest that sample dilution has an influence on NB stability and lifetime. Hence, the stability of NBs after various dilutions factors was investigated. NB samples were prepared and diluted at varying dilution factors (1–50, where 1 is stock concentration) and their concentration measured at *t* = 0 and 60 min. Concentrations after 60 min, [*C*_*t*=60_] were adjusted for their dilution factor and normalized to their initial values, [*C*_*t*=0_], to allow comparison between each of the three NB samples which had different initial NB concentrations. Results are shown in Figure 5. Across all samples (I, II, and III), [*C*_*t*=60_]/[*C*_*t*=0_] decreased with increasing dilution factor, hence suggesting that dilute NB samples are less stable. Comparison of stability between samples showed that larger NBs (i.e., sample I) have increased stability across all measured dilutions, compared to sample II and III, in agreement with the Laplace theory, which would predict stability should go as I > II > III.

NB samples consist of a mixed population of gas-filled bubbles and aqueous-filled liposomes. As such, dilution will influence the concentration of both NBs and liposomes in solution. Recently, Segers et al.^62,63^ show that the use of higher lipid concentrations, and hence higher liposome concentrations, can aid short-term and long-term MB stability. The presence of liposomes between closely approaching bubbles can provide additional colloidal and surface forces, reducing coalescence probability. In our system, the total “free” concentration (i.e., those forming liposomes, not NBs) is initially assumed to be 40 μg/mL for all samples (Methods S2) and is defined as “free lipid concentration”, as shown in Figure 5a. This parameter is analogous to dilution and hence further corroborates a link between higher lipid concentrations and NB lifetime.

Molecular dynamics simulations by Weijs et al.^64^ predicted that higher NB concentrations, and hence smaller interbubble distances, can enhance NB lifetime, providing a shield gas to diffusion. While the initial bubbles diameters, *d*, and interbubble spacings, *l*, are much smaller than those studied in our system (*d* = 3.64 nm, *l* = 15, 30 nm), assuming that their results hold for all sizes of NBs, it can be assumed there is some critical ratio between interbubble spacing and bubble diameter (*l*_0_/*d*) that will improve NB stability. It was found that for all conditions in which *l* = 15 nm (i.e., *l*/*d* = 4.12), the bubbles were shown to be stable. For those in which *l* = 30 nm (i.e. *l*/*d* = 8.24) bubbles were unstable unless a large quantity of gas was dissolved in the surrounding medium.

Further analysis of the data presented in Figure 5a, to consider the ratio of interbubble distance and diameter (*l*/*d*) is presented in Figure 5b, in which normalized NB concentration is shown after storage for 60 min for 9.5 < *l*/*d* < 51.4. Data were fit using an exponential decay function and extrapolated to interpret behavior at *l*/*d* values outside the measured range and below the minimum achievable interbubble spacing, which was restricted by the maximum initial NB concentration. As in Weijs et al., the interbubble distance, *l*, was defined as the distance between the center of each particle, and hence *l* = *n*^–1/3^, where *n* is volume density of the particles. Across all samples, NB lifetime decreases with increased *l*/*d*, in agreement with the proposed model. Interestingly, sample III (the smallest NB sample) displays enhanced stability at increased *l*/*d* values compared to samples I and II, which follow a similar trend. For example, for NB stability of sample III over 60 min (i.e., normalized NB concentration = 100%), an *l*/*d* value of 15.4 would be required, compared to 11.1 and 11.3 for samples I and II, respectively. Hence, for sample III, interbubble spacing alone cannot explain the increased stability. This may be attributed to a higher free lipid concentration, in which sample III has the highest free lipid concentration at matched NB concentrations, approximately 5 and 3 times greater than samples I and II. We believe these data suggest that both interbubble distance and free lipid concentration influence NB lifetime and stability and are important parameters that should be accounted for in future therapeutic NB studies.

While our NBs are stable for ∼60 min, dependent on their size and concentration, the rapid decay of concentration at lower lipid concentration and NB concentration would need to be improved for translation into *in vivo* use. Furthermore, a relatively high concentration of NBs was required to observe enhanced uptake, orders of magnitude higher than would be expected for MBs. Many studies have incorporated combinations of nonionic poloxamers,^7^ cross-linked polymers,^25^ and anionic lipids^29,65^ into the NB shell to increase lifetime. Traditionally these methods act to either reduce surface tension to prevent dissolution or reduce bubble coalescence via electrostatic repulsion. A recent theory proposed by Tan et al.^67^ suggests that the incorporation of ionic-lipids, and hence NBs possessing a nonzero ζ potential, may provide an electrostatic pressure which acts to counter the Laplace pressure of a collapsing bubble. As such, our use of NTA to successfully characterize NBs is promising for the future development of stable NBs, opening a range of studies to investigate the effect of varying NB shell composition on stability and *in vitro* and *in vivo* performance.

## Conclusion

Accurate characterization of NB size and population has proved challenging in the research community and may limit their transition into clinical studies. Here, we used a commercially available NTA system to determine the size and concentration of NBs only, in a mixed population containing NBs and liposomes. By considering the difference in optical properties between gas-filled bubbles and aqueous-filled liposomes and the associated change in intensity of scattered light, use of a low camera level allowed the measurement of exclusively highly scattering particles. The evidence that these particles were gas-filled and acoustically active was marked by a decrease in NB concentration after exposure to a high intensity US destruction pulse. NBs of varying size were then isolated via centrifugation, and confocal fluorescence microscopy was used to quantify NB and US mediated uptake of fluorescent dextran into SW480 cancer cells. The uptake enhancement was dependent on NB concentration, but unexpectedly at matched NB concentrations, the smallest NB sample outperformed the intermediate sample and had similar uptake performance to the largest NB sample.

To understand why, the lifetime of NBs was measured. It was found that at matched NB concentration, the smallest NB sample demonstrated enhanced stability. This may be due to an increased lipid concentration during measurement, in agreement with a previously proposed theory increased free lipid concentration can reduce the probability of coalescence. Another study based on molecular dynamics simulations suggests that small interbubble distances can increase NB stability and there exists critical interbubble distance at which NBs are found to be stable. This predicted threshold was concurrent with our results across all three NB sizes and the concentration ranges that were measured. As such, the importance of fully characterizing a therapeutic NB based system is highlighted in which key physical differences between samples have an influence on their stability, and hence their therapeutic performance.

## Notes

Data used in the figures of this paper are available at https://doi.org/10.5518/1257.

## Supporting Information Available

Nanoparticle Tracking Analysis calibration data, demonstrating the ability to correctly identify large particles; 2D K-Wave acoustic simulations to predict the maximum pressures recorded within the microfluidic channel; effect of NB on-chip incubation on sonoporation/uptake; example of the image processing used to determine total fluorescence intensity, indicative of total Dextran uptake post sonoporation of SW480 cells; NTA data of Sample I NBs (RCF = 100 g) and the liposome precursor solution, with both populations having a modal size of 90 nm, and nearly indistinguishable from each other; The effect of varying Camera Level on NTA image acquisition during measurement of the pre-cursor liposome solution at a lipid concentration of 20 μg/mL, similar to NB measurements; Volume-weighted population size distributions for NBs isolated via centrifugation; Brightfield microscopy images of NB samples; Predicted NB population distributions at the top of the microfluidic device (channel height = 400 μm), and in contact with the cell monolayer, after a 60 minute on-chip incubation period; Plots showing the trend between Total Fluorescence Intensity and predicted surface density of nanobubbles at the top of the microfluidic channel; NB stability measured in situ on the NTA system at a concentration of ∼109 NBs/mL; Table of parameters adjusted by varying camera level on the NTA system; HIFU Destruction Methodology; Free lipid concentration calculation;(PDF)
