# Supplementary material for: The Influence of Nanobubble Size and Stability on Ultrasound Enhanced Drug Delivery
Source: Langmuir. 2022 Nov 2;38(45):13943–54. doi: 10.1021/acs.langmuir.2c02303 (PMC9671049; doi:10.1021/acs.langmuir.2c02303)
Supplement: Supplementary file 1 — la2c02303_si_001.pdf [file la2c02303_si_001.pdf]

The Influence of Nanobubble Size and Stability on Ultrasound Enhanced Drug Delivery

Damien V. B. Batchelor [a], Fern J. Armistead [a], Nicola Ingram [b], Sally A. Peyman [a], James R. McLaughlan [b, c], P. Louise Coletta [b], Stephen D. Evans [a]

[a] Molecular and Nanoscale Physics Group, School of Physics and Astronomy, University of Leeds, LS2 9JT, United Kingdom

[b] Leeds Institute of Medical Research, Wellcome Trust Brenner Building, St James's University Hospital, Leeds, LS9 7TF, United Kingdom

[c] Faculty of Electronic and Electrical Engineering, University of Leeds, LS2 9JT, United Kingdom

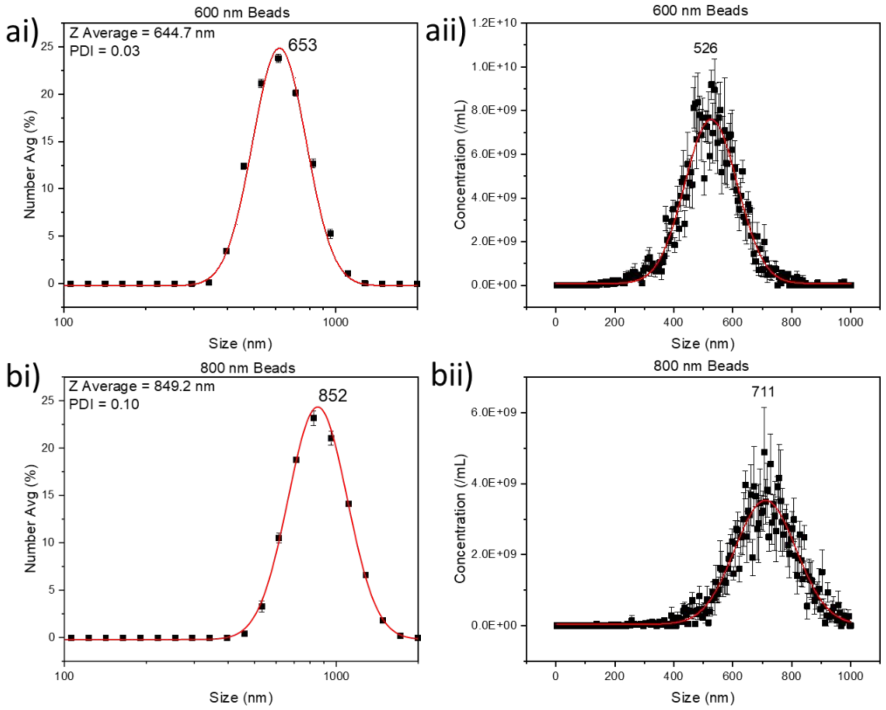

Figure S1 – Nanoparticle Tracking Analysis calibration data, demonstrating the ability to correctly identify large particles. a) Population distribution of NIST standard 600 nm polystyrene spheres measured via i) DLS (modal size = 653 nm) and ii) NTA (modal size = 526 nm). b) Population distribution of NIST standard 800 nm polystyrene spheres measured via i) DLS (modal size = 852 nm) and ii) NTA (modal size = 711 nm). The two samples had predicted concentrations of 3.73 x 10^{11} /mL and 7.67 x 10^{11} /mL for 600 nm and 800 nm beads respectively. Values acquired by the NTA system were (3.53 ± 0.14) x 10^{11} /mL and (6.00 ± 0.40) 10^{11} /mL respectively, close to the theoretical values.

Figure S2

We present 2D K-Wave acoustic simulations to predict the maximum pressures recorded within the microfluidic channel. The table below shows the material properties used for the simulations in which the gel pad, contents of the microfluidic channel and water bath were modelled as water. The top and bottom layers of the microfluidic chip were modelled as a polymer, with density and speed of sound similar to that of other commonly used polymers. (Https://link.springer.com/content/pdf/10.1134/S1995421218030152.pdf)

Material	Density (kg/m^2)	Speed of Sound (m/s)
Water	1000	1480
Polymer	1700	2000

Results of these are shown in the figure below, in which a maximum recorded pressure of 1990 kPa is recorded at the cell monolayer (x = 21 mm), likely due to reflections within the channel and the formation of standing waves. Whilst these are crude 2D simulations, it is likely that the maximum pressure recorded on-chip exceeds the incident peak negative pressure, calibrated in the free field (900 kPa).

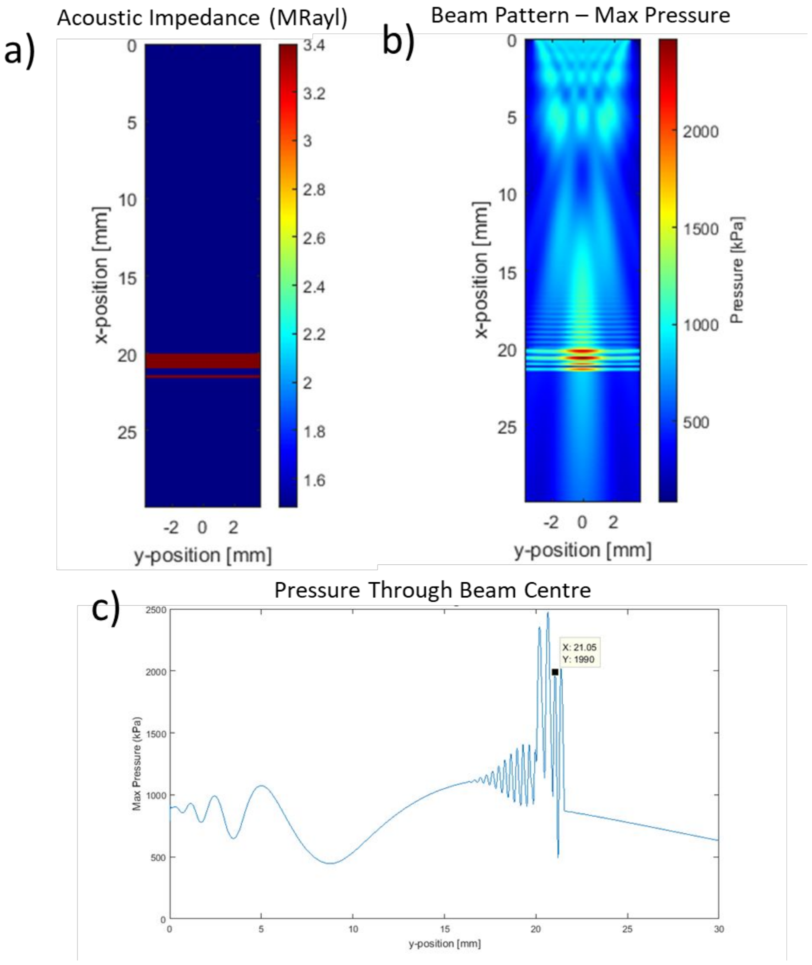

Figure S2 - Acoustic simulations of reflections within the microfluidic chip, performed using K-Wave acoustic simulation toolbox in MATLAB. a) Modelled acoustic impedances in the simulation, where ultrasound is in the positive x-direction. b) Beam pattern representing the maximum pressure during transmission with the following parameters: f_0 = 2.25 MHz, 22 cycles, 900 kPa peak negative pressure. c) Plot showing the maximum pressure recorded through the centre of the beam profile, donating the x-position where the cell monolayer is presented (x = 21 mm).

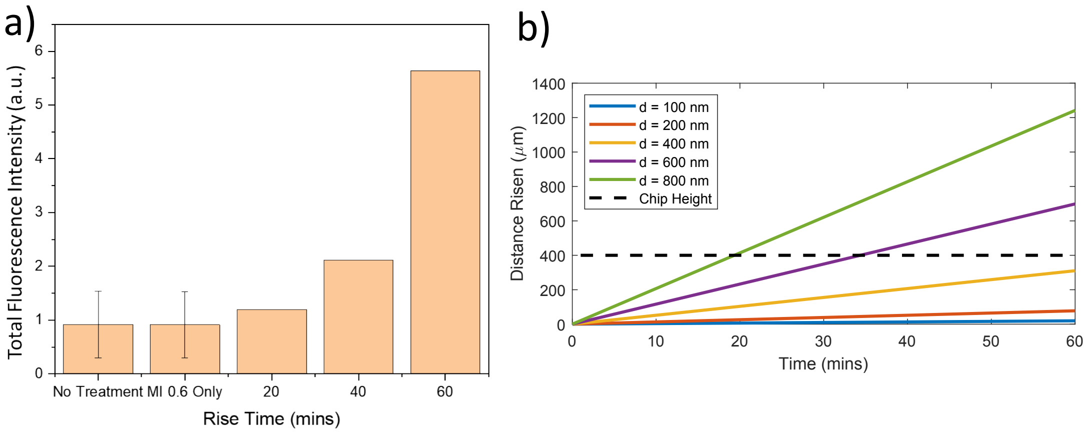

Figure S3 – (a) Effect of NB on-chip incubation on sonoporation/uptake. NBs ($2x10^{10}$ /mL) were incubated on chip for 20, 40 and 60 mins prior to insonation. b) Predicted distance risen for bubbles of varying diameter, d, over 60 minutes. Dashed line represents the channel height in the microfluidic device, and hence the time taken for a bubble at the bottom of the channel to rise to the top, and remain in contact with the cell monolayer.

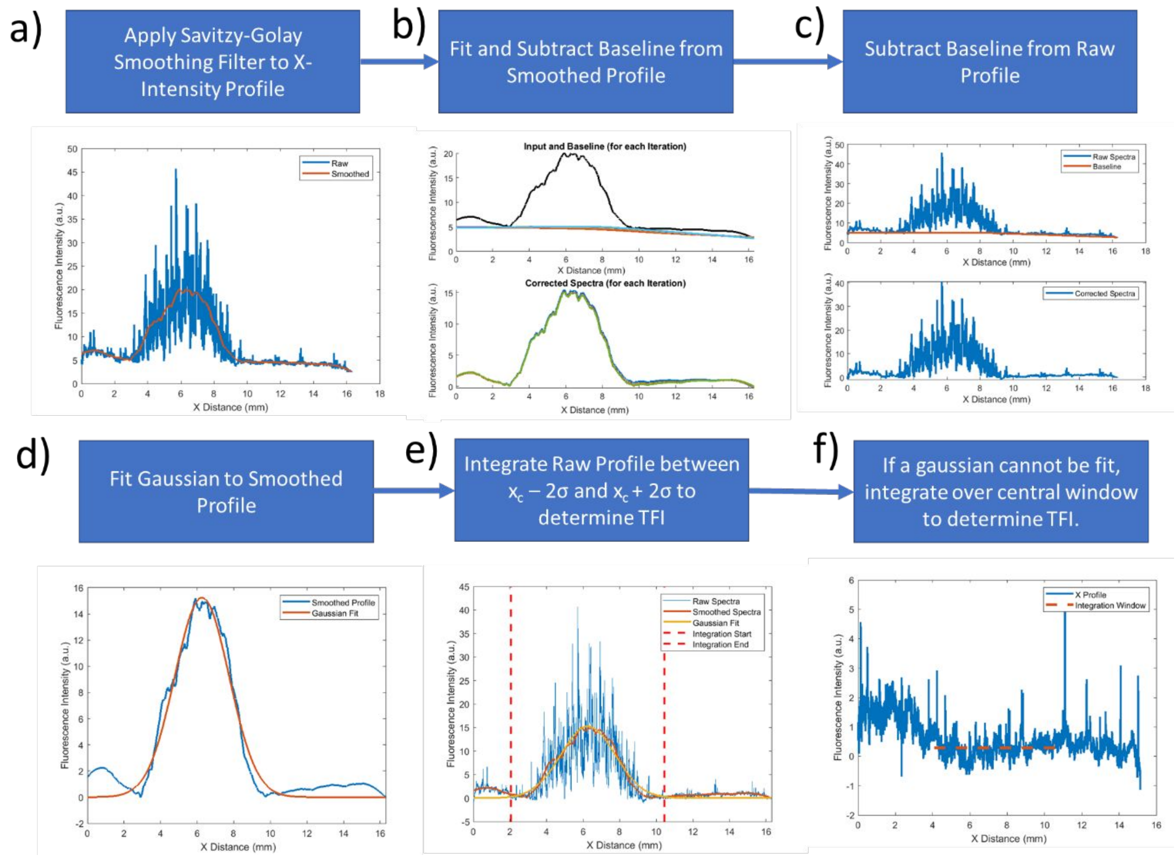

Figure S4 – Example of the image processing used to determine total fluorescence intensity, indicative of total Dextran uptake post sonoporation of SW480 cells. a) A Savitzy-Golay smoothing filter is applied with a window of 2 mm. b) A baseline is fit and subtracted from the smoothed profile. c) The baseline is subtracted from the raw, unsmoothed profile. d) A gaussian function is fit to the smoothed profile to determine the peak centre, x_c, and standard deviation σ. e) The raw, baseline corrected profile is then integrated between $x_c - 2\sigma$ and $x_c + 2\sigma$ to determine the total fluorescence intensity. f) An example shown for a profile with no clear uptake (i.e gaussian fitting was unsuccessful). In this case, a central window of 7 mm in width is integrated to determine total fluorescence intensity.

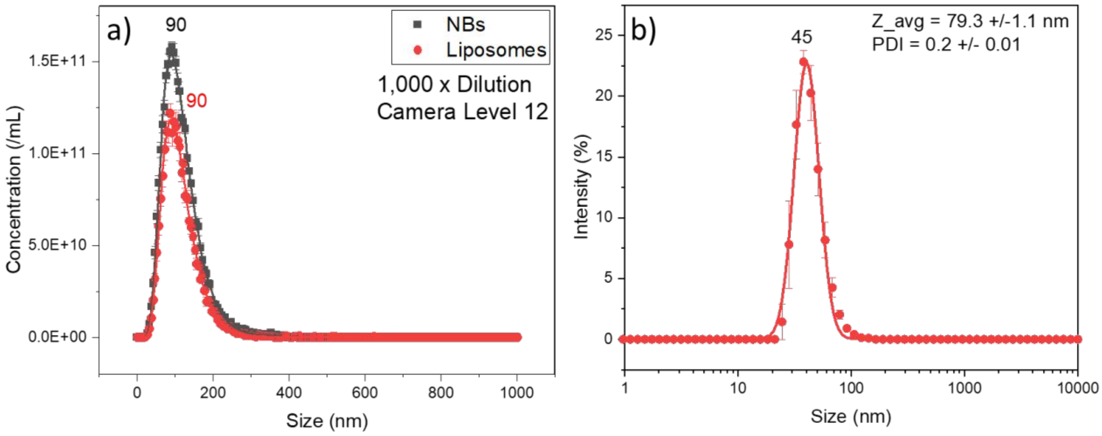

Figure S5 – NTA data of Sample I NBs (RCF = 100 g) and the liposome precursor solution, with both populations having a modal size of 90 nm, and nearly indistinguishable from each other. Both samples were measured at 1,000 x dilution (e.g. approximate lipid concentration of 20 ug/mL, corresponding to a 100 x dilution from stock concentration) and with a Camera Level 12, correlating to camera exposure time. NTA measurements of the liposomes are also in agreement with DLS measurements (b), showing the presence of a monodisperse population of particles with modal size of 45 nm and Z-Average of 79.3 nm.

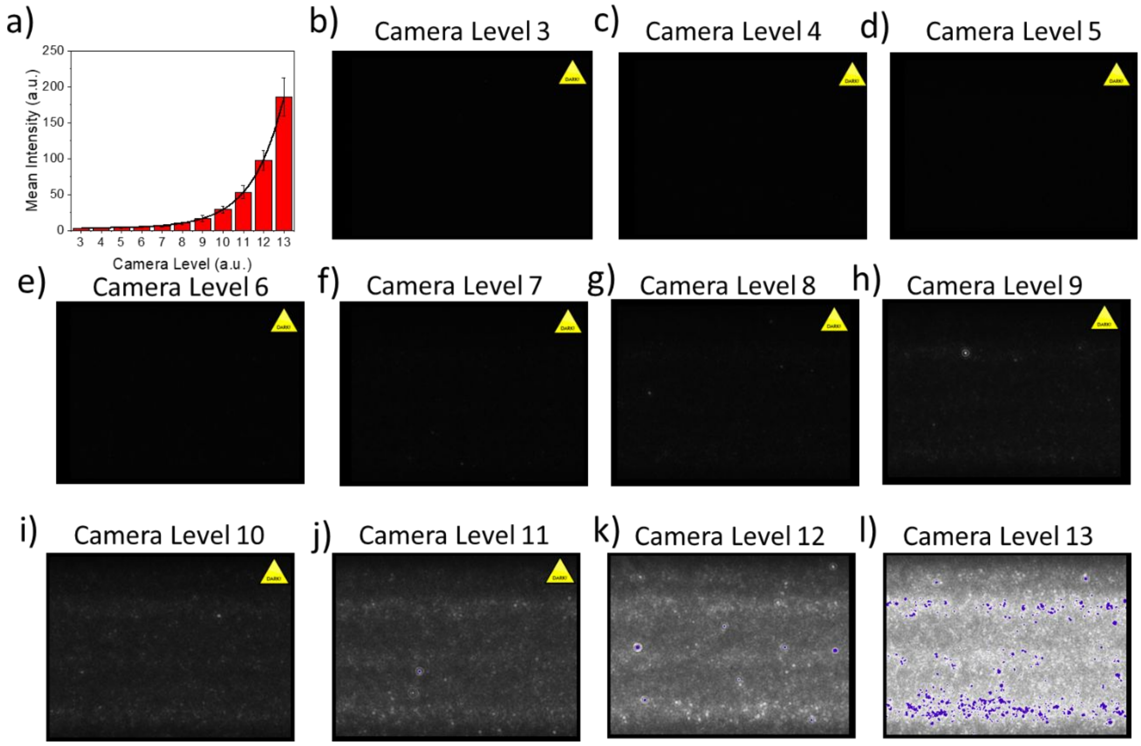

Figure S6 – The effect of varying Camera Level on NTA image acquisition during measurement of the pre-cursor liposome solution at a lipid concentration of 20 µg/mL, similar to NB measurements. A plot of mean intensity (a) in a central area of each image (b-l) for camera levels varying between 3-13, showing no detectable scattering from liposomes until the camera level is > 9, in comparison to typical values of 3-5 used for NB measurements. Hence this shows that at lower camera levels, only NBs are detectable. Further, this also shows exponential relationship between camera level and image intensity using the NTA system.

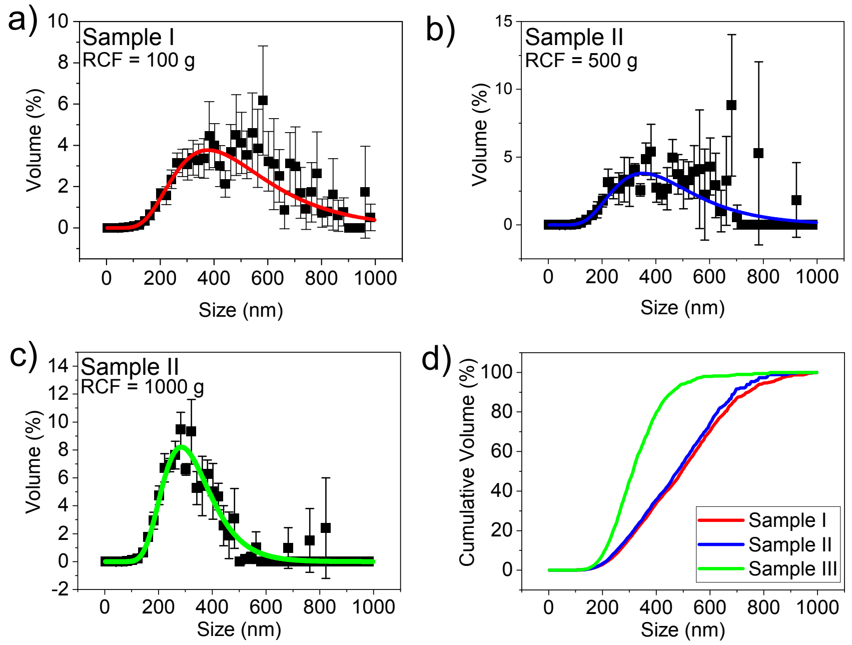

Figure S7 – Volume-weighted population size distributions for NBs isolated via centrifugation at (a) 100 g, (b) 500 g and (c) 1,000 g. d) Cumulative volume-weighted size distribution.

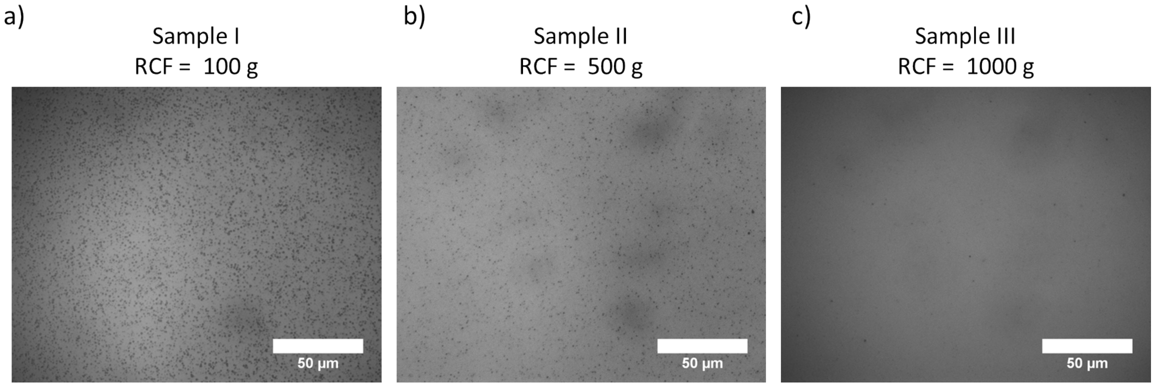

Figure S8 – Brightfield microscopy images of NB samples a) I, b) II and c) III at their yield concentrations, showing a decrease in optically visible bubbles with increasing RCF as larger bubbles are removed during the isolation process.

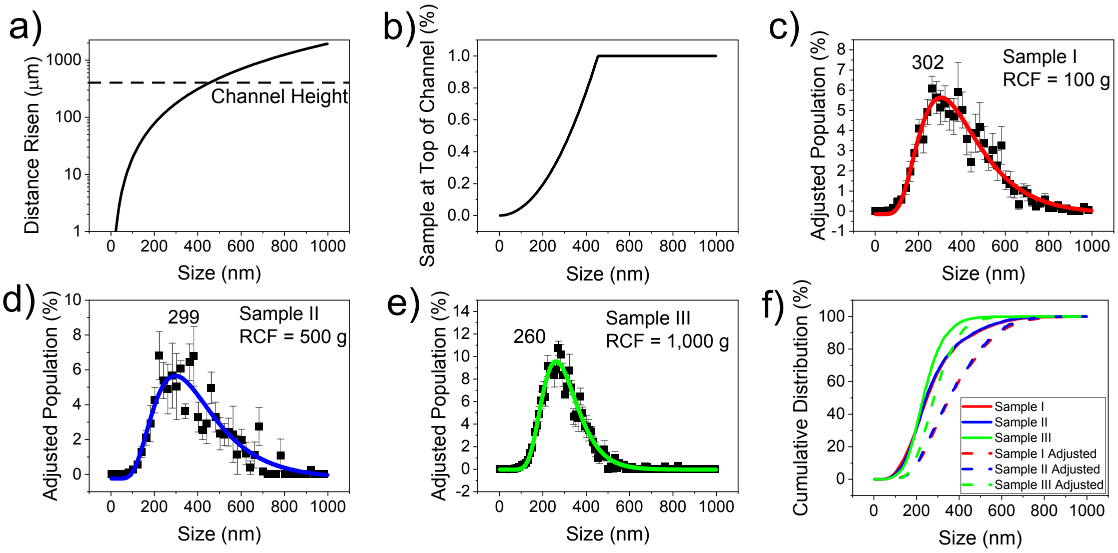

Figure S9 – Predicted NB population distributions at the top of the microfluidic device (channel height = 400 μm), and in contact with the cell monolayer, after a 60 minute on-chip incubation period. a) Predicted rise distance for NBs of varying size over 60 min. b) The proportion of NBs of varying size that will reach the top of the channel after 60 minutes. c-e) Predicted NB populations at the top of the channel after 60 min for Sample I (RCF = 100 g), II (RCF = 500 g) and III (RCF = 1,000 g). For clarity, only every 4th data point is shown. e) Cumulative distribution functions for each NB sample compared between the initial population and the adjusted population at the top of the channel.

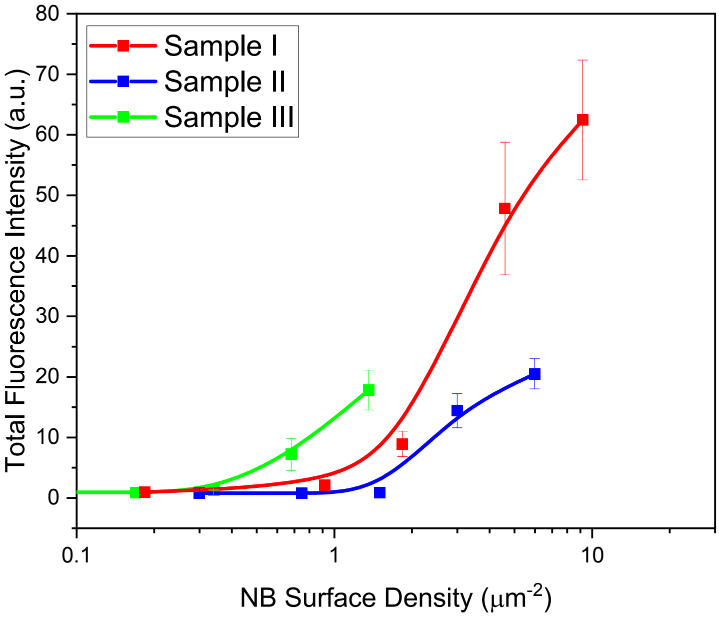

Figure S10 – Plots showing the trend between Total Fluorescence Intensity and predicted surface density of nanobubbles at the top of the microfluidic channel and hence in contact with the cell monolayer, after a 60 minute incubation and rise time. Data was fit with a spline to guide the eye.

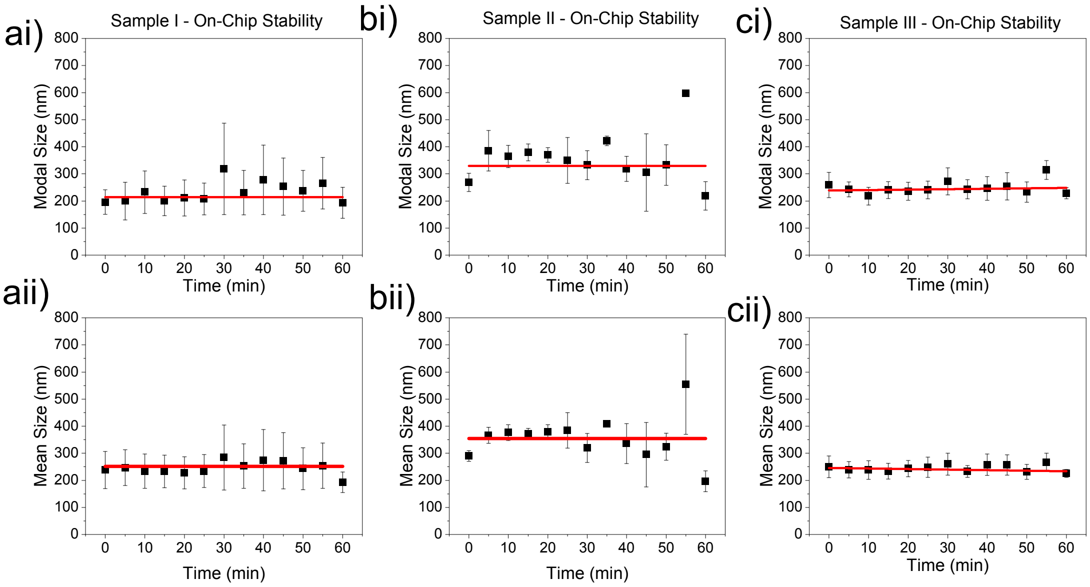

Figure S11 – NB stability measured *in situ* on the NTA system at a concentration of ~10^9 NBs/mL for NB Sample I (a), II (b) and III (c) showing their modal (i) and mean (ii) sizes over 60 min. All results were repeated in triplicate and fit using a linear fit.

Camera Level	Camera Gain (a.u.)	Shutter (ms)	Camera Histogram Upper Limit
**1**	15	0.1	16380
**2**	15	0.18	16380
**3**	15	0.33	16380
**4**	15	0.58	16380
**5**	15	1.13	16380
**6**	15	2.15	16380
**7**	15	4.13	16380
**8**	15	7.93	16380
**9**	15	15.18	16380
**10**	73	17.4	16380
**11**	146	22.25	16380
**12**	146	30	11529
**13**	219	30.8	8235
**14**	366	31.48	6588
**15**	366	30.15	3294
**16**	512	32.5	2470

Table S1 – Table of parameters adjusted by varying camera level on the NTA system. Camera gain corresponds to a unitless parameter defining gain applied to the CCD camera, shutter is the camera shutter time in ms and camera histogram upper limit is the maximum value of the histogram of pixel intensity.

Methods S1 – HIFU Destruction Methodology.

A single-element HIFU transducer was used for US-mediated NB destruction. A 1.1 MHz center frequency HIFU transducer (H-102, Sonic Concepts, USA) was used for all HIFU experiments. The transducer was connected to a +55 dB power amplifier (A300, E&I Ltd, USA) via an impedance matching circuit. A computer-controlled function generator (33220A, Agilent, USA) was used to provide sinusoidal burst cycles to the transducer. The free-field pressure was measured using a membrane hydrophone (Precision Acoustic Ltd, Dorchester, UK) with a 400 µm sensitive element, calibrated by the National Physics Laboratory (Middlesex, UK). A peak negative pressure of 4.83 MPa was used, based from their free-field calibrations with errors of ±0.1 MPa. Exposures had a total duration of 5 s, a duty cycle of 50 % and a pulse repetition frequency of 1 kHz. A total sample volume of 1 mL held in a micro-centrifuge tube was placed in the central opening of a coupling cone containing degassed Milli-Q water, such that the sample is located within the focal point of the HIFU beam.[1]

Methods S2 – Free Lipid Concentration

Initially, the produced MB sample will have a lipid concentration of 2 mg/mL. This sample is then diluted 10 x (i.e. 0.2 mg/mL) prior to isolation of NBs. Based off the 95:5 molar ratio of DPPC (M_w=734 g/mol) and DSPE-PEG2000 (M_w = 2805 g/mol), the MB solution will contain 1.44×10^{17} lipid molecules in total. Assuming a lipid head group area of 0.5 nm^2 and MB size of 1 μm at a concentration of 5×10^9 /mL (after dilution), MBs would contribute to 6.28×10^{16} lipids/mL. This sample would also be expected to contain a proportion of NBs. Assuming this NB population is identical in size and concentration to that of Sample I, this would account for 5.12×10^{16} lipids/mL. Here, the remaining lipids (2.98×10^{16} lipids/mL) can be accounted for by "free" liposomes in solution. This value of liposomal lipids would be expected to remain constant throughout the NB isolation process, as the vertical distance travelled during centrifugation at 1,000 g over 5 min is ~10^{-6} m is negligible (~ 50 μm).

When considering the contribution of free liposomes/lipid to NB stability by preventing coalescence via steric effects, it is only this concentration that is important. Here, the free lipid concentration of 2.98×10^{16} lipids/mL is equivalent to a mass concentration of 0.04 mg/mL, which will be consistent throughout all NB samples.
